# Virulence Determinants in *Staphylococcus aureus* Clones Causing Osteomyelitis in Italy

**DOI:** 10.3389/fmicb.2022.846167

**Published:** 2022-03-03

**Authors:** Fernanda Pimentel de Araujo, Mattia Pirolo, Monica Monaco, Maria Del Grosso, Simone Ambretti, Donatella Lombardo, Tiziana Cassetti, Raffaele Gargiulo, Eleonora Riccobono, Paolo Visca, Annalisa Pantosti

**Affiliations:** ^1^Department of Infectious Diseases, Istituto Superiore di Sanità, Rome, Italy; ^2^Department of Science, Roma Tre University, Rome, Italy; ^3^Department of Veterinary and Animal Sciences, Faculty of Health and Medical Sciences, University of Copenhagen, Frederiksberg, Denmark; ^4^Unit of Microbiology, Policlinico S. Orsola, IRCCS Azienda Ospedaliero-Universitaria di Bologna, Bologna, Italy; ^5^Unit of Clinical Microbiology, S. Agostino-Estense Hospital Baggiovara, AUSL Modena, Modena, Italy; ^6^Department of Experimental and Clinical Medicine, University of Florence, Florence, Italy; ^7^Santa Lucia Foundation (IRCCS), Rome, Italy

**Keywords:** *Staphylococcus aureus*, virulence genes, osteomyelitis, clones, antibiotic resistance, whole genome sequencing

## Abstract

*Staphylococcus aureus* is the most common pathogen causing osteomyelitis (OM). The aim of this study was to explore the clonal complex (CC) distribution and the pattern of virulence determinants of *S. aureus* isolates from OM in Italy. Whole-genome sequencing was performed on 83 *S. aureus* isolates from OM cases in six hospitals. Antibiotic susceptibility tests showed that 30.1% of the isolates were methicillin-resistant *S. aureus* (MRSA). The most frequent CCs detected were CC22, CC5, CC8, CC30, and CC15, which represent the most common lineages circulating in Italian hospitals. MRSA were limited in the number of lineages (CC22, CC5, CC8, and CC1). Phylogenetic analysis followed the sequence type-CC groupings and revealed a non-uniform distribution of the isolates from the different hospitals. No significant difference in the mean number of virulence genes carried by MRSA or MSSA isolates was observed. Some virulence genes, namely *cna*, *fib*, *fnbA*, *coa*, *lukD*, *lukE*, *sak*, and *tst*, were correlated with the CC. However, different categories of virulence factors, such as adhesins, exoenzymes, and toxins, were frequently detected and unevenly distributed among all lineages. Indeed, each lineage carried a variable combination of virulence genes, likely reflecting functional redundancy, and arguing for the importance of those traits for the pathogenicity in OM. In conclusion, no specific genetic trait in the most frequent lineages could explain their high prevalence among OM isolates. Our findings highlight that CCs detected in OM isolates follow the epidemiology of *S. aureus* infections in the country. It is conceivable that any of the most common *S. aureus* CC can cause a variety of infections, including OM.

## Introduction

Osteomyelitis (OM) is an infective and inflammatory process of the bone, which can progress to osteonecrosis and bone destruction. Treatment of OM is challenging due to a variety of factors including the scarce penetration of antibiotics into the bone tissue, the biofilm-like presentation of the infection, and the increasing antibiotic resistance of bacterial pathogens. Despite the improvement in the diagnosing of OM, the incidence of OM has been increasing over the last few decades. This could be ascribed to the increase of certain risk factors such as diabetes and surgery procedures such as arthroplasty (Kurtz et al., 2012; Geraghty and LaPorta, 2019). The number of orthopaedic surgeries is constantly increasing. In the United States (US), the average orthopaedic surgical volume increased of 38% in the last 3 years (Shukla et al., 2021) and is estimated that by 2030, 572,000 hip revisions and 3.48 million knee revisions could be performed with an increase of 174 and 673%, respectively (Kurtz et al., 2012). In line with the increase of arthroplasty, the incidence of OM increased from 11.4 to 24.2 cases per 100,000 person-years in four decades in United States (Kremers et al., 2015). If a prosthetic-joint infection develops, revision surgery for removal of the implant is often required, which leads to prolonged hospitalization and a high risk of re-infection (Trampuz and Zimmerli, 2008).

*S. aureus* is the principal cause of OM, being responsible for 30–60% of cases (Karwowska et al., 1998; Grammatico et al., 2008; Byren et al., 2009; Howard-Jones and Isaacs, 2013). OM caused by *S. aureus* is associated with severe prognosis and persistent infections in approximately 40% of patients (Kremers et al., 2015). During bone infection, *S. aureus* attachment and colonization are facilitated by adhesins. The colonization of bone occurs through direct interaction of *S. aureus* with the bone cells or the extracellular matrix (ECM). After bone colonization, *S. aureus* can grow as biofilm and produce toxins, which facilitate persistence and dissemination of the infection in the host, respectively (Patti et al., 1994; Tuchscherr et al., 2019). The success of *S. aureus* in bone infections is related to a multitude of virulence factors involved in critical steps of the pathogenesis, including adhesion to surfaces, invasion of host tissues, evasion of the immune system, and biofilm formation (Ricciardi et al., 2018).

*S. aureus* strains from different sources carry various combinations of virulence genes (Montanaro et al., 1999; Peacock et al., 2002; Campoccia et al., 2008), and some studies were conducted to identify and characterize the most frequent *S. aureus* clones cause of OM (reviewed by Pimentel de Araujo et al., 2021). Pandemic lineages, including CC5, CC8, CC22, CC30, and CC45, were the most common in OM, and their distribution greatly differed among the countries reflecting the local epidemiology of *S. aureus* and the MSSA heterogeneity (Pimentel de Araujo et al., 2021). However, the characterization of *S. aureus* clones causing bone and joint infections in Italy is limited to only few studies (Campoccia et al., 2008; Montanaro et al., 2016).

It is unknown to date if the ability to cause OM of some lineages is correlated with the presence of certain combination of virulence factors, or it is only a reflection of the local epidemiology of clones. The purpose of this study was to identify the pattern of virulence determinants and the clonal distribution of *S. aureus* isolates from OM in Italy, by using whole-genome sequencing (WGS) for a detailed genetic characterization.

## Materials and Methods

### *Staphylococcus aureus* Collection

From February 2019 to February 2020, a total of 83 non-duplicate *S. aureus* isolates were collected from patients with OM, in six hospitals in two Italian Regions (Tuscany and Emilia-Romagna). A positive case of *S. aureus* OM was ascertained upon identification of *S. aureus* from bone biopsy, aspirate of intraosseous abscess, intraoperative swabs, prosthetic implants, or any osteosynthesis material. *S. aureus* isolates were isolated and identified by the hospital laboratories according to the local procedures, including both MALDI-TOF and the Vitek2® system (BioMérieux, Marcy l’Etoile, France). Isolates were shipped to Istituto Superiore di Sanità (ISS, Rome, Italy) and Roma Tre University for the phenotypic and genotypic characterization, respectively. Strains have been screened for oxacillin resistance according to EUCAST protocol (version 11.0, 2021).^1^ The study was approved by the Ethics Committee of ISS (n° 0013802 18/03/2019) and the participating hospitals.

### Antibiotic Susceptibility

Antibiotic susceptibility testing was preliminary performed using the Vitek2® system (BioMérieux, Marcy l’Etoile, France) or MicroScan Walkaway (Beckman, United States) at the participating hospital laboratories, and subsequently by the broth microdilution method using commercially available microplates (MERLIN Diagnostika GmbH, Germany). Discrepant results were resolved by testing individual isolates with the disk diffusion method. Results were interpreted according to the EUCAST breakpoints (version 11.0, 2021; see footnote 1).

### DNA Isolation, Whole-Genome Sequencing, and Genotyping

Genomic DNA of the 83 *S. aureus* isolates was extracted using the QIAamp DNA Mini Kit (QIAGEN srl, Milan, Italy) according to the manufacturer’s protocol, with the only modification of the addition of 50 μg/ml of lysostaphin (Sigma Aldrich, Milan, Italy) for the lysis step. Sequencing was performed by using Illumina MiSeq (Illumina, San Diego, CA, United States). Processed FASTQ reads were *de novo* assembled using SPAdes pipeline (Bankevich et al., 2012) through the ARIES public Galaxy server.^2^ Sequence Types (ST)s, *spa* types, and Staphylococcal Cassette Chromosome *mec* (*SCCmec*) types were identified by MLST v2.0, *spa*Typer v1.0 and *SCCmec* Finder v1.2 pipelines, respectively, available at the CGE website.^3^ Clonal complex (CC) grouping was performed using pubMLST website.^4^ Antimicrobial resistance genes were searched in the assembled genomes of all isolates using ABRicate v1.0.1 and both ResFinder and CARD databases (Zankari et al., 2012; Jia et al., 2017).^5^ Positive hits were selected based on >95% nucleotide sequence identity. Point mutations in *gyrA* and *grlA* genes conferring resistance to quinolones, and in *rpoB* conferring rifampicin resistance, were searched in the assembled genomes using reference gene sequences from *S. aureus* NCTC 8325 (GenBank accession no. CP000253.1). Translated protein sequences were aligned using ClustalW in MEGA X v.10.2.1 with default parameters (Kumar et al., 2018) and inspected for known substitutions conferring resistance to quinolones and rifampicin (Aubry-Damon et al., 1998; Tanaka et al., 2000).

To screen for virulence factors, a custom database based on the allele library previously described by Strauß et al. (2016) was constructed. The database comprised 1,205 allelic variants of 122 known virulence genes (Supplementary Table S1), including those encoding exoenzymes, toxins, adhesins superantigens, capsule, regulators, and biofilm formation. The presence of virulence determinants was ascertained using ABRicate v1.0.1 and alignment results with identity scores greater than 95% were selected as positive matches.

Core genome MLST (cgMLST)-based genotyping was performed on the 1,861 target genes of the *S. aureus* cgMLST scheme (Leopold et al., 2014). Isolates showing less than 11 allelic differences in the core genome were considered as genetically indistinguishable (Leopold et al., 2014). A neighbor-joining (NJ) tree was created based on the allelic profiles of the cgMLST target genes. All analyses were conducted in SeqSphere+ v8.0.1 (Ridom GmbH, Germany).

### Statistical Analysis

Data analysis was performed in R v4.1.1. Normality distribution of virulence factors among CCs was evaluated using the Shapiro–Wilk’s method. Normally and non-normally distributed data were compared with Student’s *t*-test and Mann–Whitney-Wilcoxon’s test, respectively, with Benjamini-Hochberg’s correction for false discovery rate. Adjusted *p* ≤ 0.05 were considered as significant.

### Data Availability

WGS data for the 83 *S. aureus* isolates have been submitted to the NCBI SRA under BioProject PRJNA784720.

## Results

### Demographic Characteristics of the Patients

The main characteristics of OM patients are summarized in Table 1. Most patients were adults (mean age 60.9 ± 17.1 years, range 22–94), and 54.2% were male. Additional information was available only for a portion of the patients/isolates. The most common source of infection was orthopaedic surgery (42/54 patients, 77.7%) in the presence of a prosthetic implant (37/54, 68.5%); hematogenous OM was observed only in 11.1% (6/54) of the patients. The systemic risk factor most reported was diabetes (6/38, 15.8%). OM affected primarily lower limbs (26/39, 66.6%), followed by upper limbs and vertebrae (17.9 and 15.4% of the patients, respectively).

**Table 1:** Demographic and clinical characteristics of OM patients.

Characteristics (no. of patients with data)^a^	No. of positive patients (%)
Age (years), mean ± SD (83)	60.9 ± 17.1
Male sex (83)	45 (54.2)
Recent hospitalisation (<1 year; 38)	20 (52.6)
**Origin of infection**	
Orthopaedic surgery (54)	42 (77.7)
Hematogenous spread (54)	6 (11.1)
Others (54)	6 (11.1)
**Local risk factors**	
Implants or devices for osteosynthesis (54)	37 (68.5)
Others (54)	8 (14.8)
No risk factor (54)	9 (16.6)
**Systemic risk factors**	
Diabetes (38)	6 (15.8)
Vasculopathy (38)	2 (5.3)
Others (38)	4 (10.5)
No risk factor (38)	26 (68.4)
**Localization of infection**	
Lower limb (39)	26 (66.6)
Upper limb (39)	7 (17.9)
Vertebrae (39)	6 (15.4)

a *As provided by the hospitals*.

### Antibiotic Susceptibility

The results of antimicrobial susceptibility testing of the 83 *S. aureus* isolates are shown in Table 2. All isolates were susceptible to vancomycin, and 30.1% were resistant to methicillin (MRSA). MRSA isolates showed elevated frequencies of resistance to levofloxacin (84.0%), erythromycin (64.0%), clindamycin (64.0%) and gentamicin (32.0%; Table 2). The majority of MSSA isolates were resistant to penicillin (62.1%) and, less frequently, to levofloxacin (17.2%), erythromycin (13.8%) and clindamycin (13.8%; Table 2).

**Table 2:** Resistance genes and antimicrobial-susceptibility profile in 83 *Staphylococcus aureus* isolates from OM.

Antimicrobial class	Antimicrobial resistance gene	No. of isolates with resistant gene (%)			Antimicrobial^a^	No. of isolates with resistant phenotype (%)		
		MSSA (*n* = 58)	MRSA (*n* = 25)	All isolates (*n* = 83)		MSSA (*n* = 58)	MRSA (*n* = 25)	All isolates (*n* = 83)
β-lactams	*blaZ*	36 (62.1)	19 (76.0)	55 (66.3)	PEN^b^	36 (62.1)	25 (100.0)	61 (73.5)
	*mecA*	0	25 (100.0)	25 (30.1)	OXA	0	25 (100.0)	25 (30.1)
					FOX	0	25 (100.0)	25 (30.1)
Fluoroquinolones	*gyrA* S84L *grlA* S80F	4 (6.9)	17 (68.0)	21 (25.3)	LEV	10 (17.2)	21 (84.0)	31 (37.3)
	*gyrA* S84L *grlA* S80Y	0	3 (12.0)	3 (3.6)				
MLSB	*ermC*	2 (3.4)	10 (40.0)	12 (14.5)	ERY	8 (13.8)	16 (64.0)	24 (28.9)
	*ermA*	1 (1.7)	6 (24.0)	7 (8.4)	CLI^c^	8 (13.8)	16 (64.0)	24 (28.9)
	*ermT*	5 (8.6)	0	5 (6.0)				
Aminoglycosides	*ant(9)-Ia*	1 (1.7)	6 (24.0)	7 (8.4)	GEN	2 (3.4)	8 (32.0)	10 (12.0)
	*aph(3′)-III*	0	4 (16.0)	4 (4.8)				
	*aac(6′)-aph(2″)*	2 (3.4)	4 (16.0)	6 (7.2)				
	*ant(6)-Ia*	0	2 (8.0)	2 (2.4)				
	*aadD*	0	1 (4.0)	1 (1.2)				
Glycycycline					TGC	0	0	0
Rifampicin	*rpoB* H481N	0	3 (12.0)	3 (3.6)	RIF	1 (1.7)	5 (20.0)	6 (7.2)
	*rpoB* H481L	1 (1.7)	1 (4.0)	2 (2.4)				
Tetracyclines	*tetM*	0	2 (8.0)	2 (2.4)	TET	0	4 (16.0)	4 (4.8)
	*tetL*	0	1 (4.0)	1 (1.2)	DOX	0	4 (16.0)	4 (4.8)
	*tetK*	0	1 (4.0)	1 (1.2)				
Sulfonamides	*dfrC*	0	2 (8.0)	2 (2.4)	T/S	1 (1.7)	2 (8.0)	3 (3.6)
	*dfrG*	1 (1.7)	0	1 (1.2)				
Fusidic acid	*fusC*	0	1 (4.0)	1 (1.2)	FUS	0	1 (4.0)	1 (1.2)
Lipopeptides					DPT	0	1 (4.0)	1 (1.2)
Oxazolidinones					LIZ	0	0	0
Glycopeptides					VAN	0	0	0
					TPL	0	0	0

a CLI, clindamycin; DOX, doxycycline; DPT, daptomycin; ERY, erythromycin; FOX, cefoxitin; FUS, fusidic acid; GEN, gentamicin; LEV, levofloxacin; LIZ, linezolid; MLSB, macrolides-lincosamides-streptogramin B; OXA, oxacillin; PEN, penicillin; RIF, rifampicin; TET, tetracycline; TGC, tigecycline; TPL, teicoplanin; T/S, trimethoprim-sulfamethoxazole; VAN, vancomycin.

b Data obtained by automated system Vitek2® (BioMerieux).

c Clindamicyn inducible strains *n* = 15.

### *Staphylococcus aureus* Genotyping

**Figure 1:**
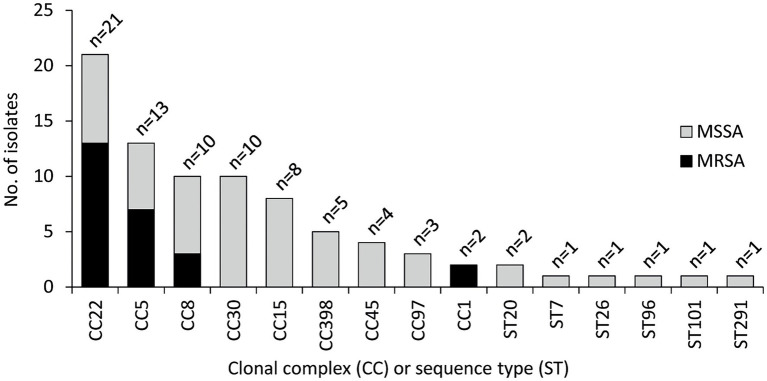
Clonal complex (CC) and sequence type (ST) distribution of 83 *Staphylococcus aureus* isolates from OM. CC22 (ST22, *n* = 20; ST3863, *n* = 1), CC5 (ST5, *n* = 9; ST105, *n* = 2; ST228, *n* = 2), CC8 (ST8, *n* = 7; ST72, *n* = 1; ST368, *n* = 1; ST789, *n* = 1), CC30 (ST30, *n* = 7; ST34, *n* = 1; ST4391, *n* = 1; ST7297, *n* = 1), CC15 (ST15, *n* = 5; ST582, *n* = 3), CC398 (ST398, *n* = 5), CC45 (ST45, *n* = 4), CC97 (ST97, *n* = 3), CC1 (ST1, *n* = 1; ST6927, *n* = 1), ST20 (*n* = 2), ST7 (*n* = 1), ST26 (*n* = 1), ST96 (*n* = 1), and ST101 (*n* = 1).

The CC distribution for MRSA and MSSA is shown in Figure 1. Overall, 53 different *spa* types were detected (Supplementary Table S3), and isolates clustered into 26 STs and nine CCs (Figure 1). CC22 was the most frequent (25.3% of the isolates), followed by CC5 (15.7%), CC8 (12.0%), CC30 (12.0%), and CC15 (9.6%). The largest variability was observed among MSSA isolates, which were distributed into 20 STs and eight CCs. CC30 and CC22 were the most frequent CCs among MSSA and MRSA, respectively, whereas CC5, CC8, and CC22 were detected among both MSSA and MRSA isolates.

**Figure 2:**
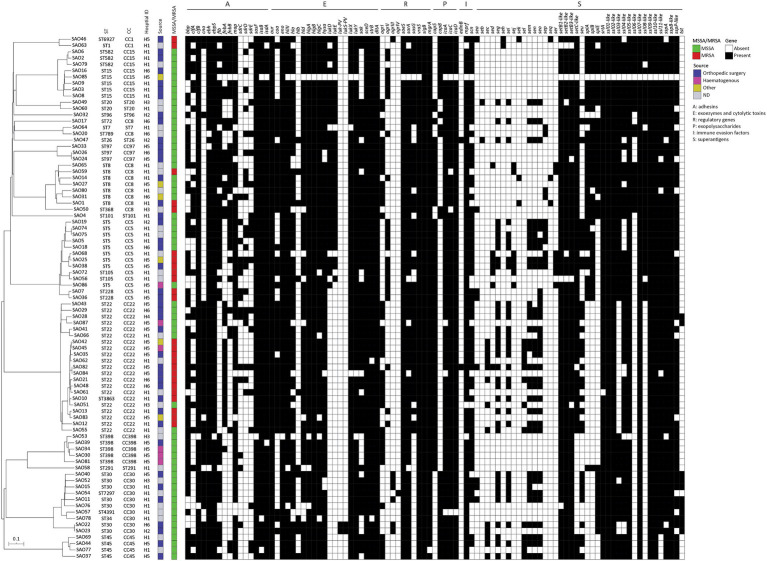
Neighbor joining (NJ) tree based on the allelic profiles of the cgMLST target genes (*n* = 1,861) of 83 *Staphylococcus aureus* isolates from osteomyelitis in Italy, and associated heat-map of *in silico* detected virulence genes (indicated on top).

The phylogenetic relationship based on cgMLST of the isolates is shown in Figure 2. Overall, phylogeny follows the ST-CC groupings and reveals a non-uniform distribution of the isolates from the six hospitals, which appeared intermingled throughout the phylogeny and did not cluster according to the geographic origin (see hospital ID in Figure 2). Interestingly, five cgMLST clusters of genetically indistinguishable isolates were observed (no. of allelic differences ≤3), containing a total of 10 isolates (two isolates per cluster), both MSSA and MRSA (Supplementary Table S2). In three cases, isolates were collected from the same hospitals (SAO42/SAO45, SAO74/SAO75, and SAO82/SAO84), whereas two clusters contained isolates originating from different hospitals (SAO22/SAO23 and SAO24/SAO26; Supplementary Table S2) in the same Italian region (H2, H5, and H6; see Supplementary Table S3).

### Detection of Virulence-Related Genes

A dataset of the 122 virulence-related genes (Figure 2; Supplementary Table S3) was used to screen 83 *S. aureus* strains from OM. An average of 53.7 ± 7 virulence-related genes was detected in the whole collection (coefficient of Variation = 14.5%); of these, 20 genes were detected in ≥95% of the isolates and eight in ≤10%. The most frequent genes encoded different categories of virulence factors, namely haemolysins (*hlIII*, *hlgA*, and *hlgBIII*), proteases (*lukX* and *lukY*), several staphylococcal superantigen-like proteins (*ssl01-like*, *ssl02-like*, *ssl05-like*, *ssl09-like*, and *ssl10-like*), exoenzymes (*srtA*), cell wall anchored proteins (CWA) endowed with adhesive properties (*ebh*, *ebps*, *sasF*, *isaB*, and *isdA*), and regulatory genes (*saeS*, *vraS*, *sigB*, and *mgrA*; Figure 2). Genes encoding exotoxins such as Panton–Valentine leukocidin (*lukS-PV* and *lukF-PV*), epidermal cell differentiation inhibitor (*edinB*), and superantigens (*seb*, *seh*, *sej*, *sek*, *seq*, and *ser*) were less frequently detected.

**Figure 3:**
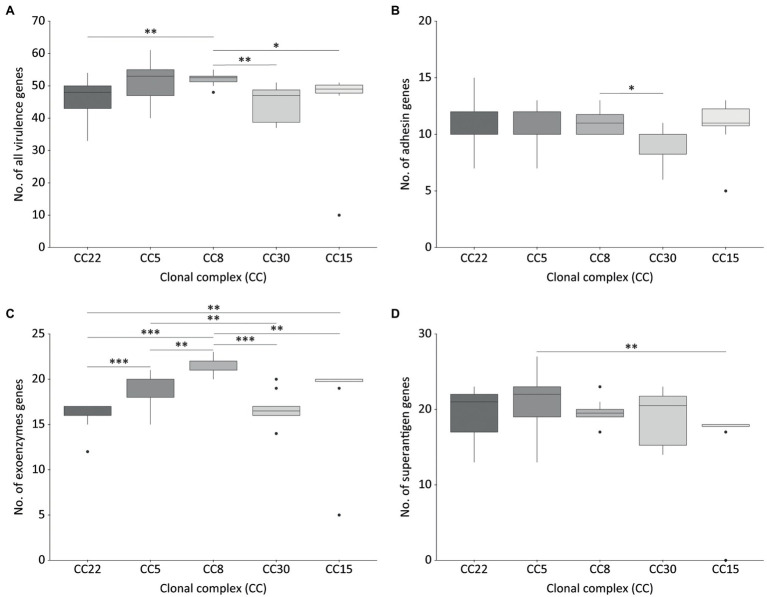
Virulence-related genes detected in isolates belonging to the most frequent *Staphylococcus aureus* clonal complexes (CC) causing OM. All virulence genes **(A)**, adhesin genes **(B)**, exoenzymes genes **(C)**, and superantigen genes **(D)**. CC22, *n* = 21; CC5, *n* = 13; CC8, *n* = 10; CC30, *n* = 10; CC15, *n* = 8. The significance of the differences in the number of virulence factors between CCs was assessed using the Mann–Whitney-Wilcoxon’s test (non-normally distributed data) for all pairwise comparisons with the exception of CC15 vs. CC30, for which Student’s *t*-test was employed (normally distributed data). Boxes denote the second and third quartiles, vertical lines (whisker) the smallest and largest values of the first and fourth quartiles, with outliers marked by dots. ^*^p < 0.05, ^**^p < 0.01, and ^***^p < 0.001.

No significant difference in the mean number of virulence genes carried by MRSA or MSSA isolates was observed. Overall, CC8 carried a significantly higher number of virulence genes than CC15, CC22, and CC30 isolates (Figure 3A).

### Adhesins

Genes coding for microbial surface components recognizing adhesive matrix molecules (MSCRAMMs) and other CWA proteins were similarly distributed among all major *S. aureus* CCs, except for CC30 which showed significantly lower adhesin genes than CC8 (Figure 3B; Supplementary Table S3). The adhesin genes *clfB*, *ebh*, *ebpS*, *sasF*, *isaB*, and *isdA* were widespread in the whole collection, being present in ≥95% of the isolates, while the fibronectin-binding protein genes (*fnbA*, *fnbB*) and collagen adhesin gene (*cna*) were detected in nearly half of the strains (range 43.7–47.1%), and in different combinations. Some adhesins genes such as *cna* and *fib* were differently associated with CC (*p* < 0.001). The *cna* gene was detected in CC1, CC22, CC30, CC45, and CC398, *fib* in CC1, CC5, CC8, CC15, CC30, CC45, CC97, and CC398.

### Exoenzymes and Toxins

Virulence factors with enzymatic or lytic properties responsible for nutrient acquisition, bacterial survival and dissemination were detected in all CCs. Haemolysins and lytic toxins (*aur*, *eno*, *hlIII*, *hlgA*, *hlgB*, *hlgC*, *hysa*, *sceD*, *strB*, and *dltA*) were present in most of the strains (≥90%). The presence of some exoenzymes genes as *coa*, *lukD*, *lukE*, and *sak* was correlated with the CC (*p* < 0.001). Isolates belonging to CC8, CC22, CC30, CC45, and CC97 were associated with *coa*. The *lukD* and *lukE* genes were associated with CC8 and CC97. The *sak* gene was detected in more than 80% of CC5, CC8, CC22, and CC97. CC8 harbored a significantly higher number of exoenzyme and toxin genes compared to the other CCs (Figure 3C; Supplementary Table S3). Panton-Valentin leukocidin (PVL) was detected in only two isolates (2.4%) from different hospitals and belonging to CC30, which clustered together within the cgMLST phylogeny and showed a similar virulence pattern (Figure 2).

### Superantigens

The enterotoxin gene cluster *egc* (*seg*, *sei*, *sem*, *sen*, *seo*, and *seu*) was detected in 31.3% of the isolates, mostly belonging to CC22, CC30, and CC5. The cluster was absent in all CC398 isolates and all but one CC8 isolate. Among the predominant CCs, a significant difference in the number of superantigen genes was observed between CC5 and CC15 (Figures 2, 3D; Supplementary Table S3). The staphylococcal superantigen-like genes were present in all CCs with minor differences. The toxic shock syndrome toxin gene (*tst*) was associated with CC30 and was detected in 13.3% of the isolates, all belonging to CC30 and CC22.

### Biofilm and Capsular Genes

Capsular genes were detected in all the isolates and the most frequent capsular type was type 5 (*cap5*; 65.0%), associated with CC5, CC8, CC22, CC97, and CC398. Capsular type 8 (*cap8*) was found in CC1, CC15, CC30, and CC45. The complete *icaACD* locus encoding genes involved in biofilm production was detected in 77 out of 83 strains (92.7%). Two strains belonging to CC30 and CC15 did not carry any of the *ica* genes, while four strains belonging to CC8 (two isolates), CC22, and CC97 (one isolate each) were negative for at least one gene of the locus.

### Regulatory Genes

The accessory gene regulator system (*agr*) responsible for the regulation and expression of toxins and exoenzymes and biofilm was detected in 86.7% of the isolates (72/83). The most frequent *agr* type was *agrI* (48.2% of the isolates), associated with CC8, CC22, CC45, CC97, CC398, and CC30 (only one strain); *agrII* was most frequently detected among CC5, CC15 and less in CC8 and CC22; *agrIII* was detected in CC1 and CC30, and *agrIV* in a single isolate belonging to CC30.

### Resistance Genes

The *mecA* gene was detected in 30.1% of the isolates belonging to CC22, CC5, CC8, and CC1. The majority of MRSA isolates carried the SCC*mec* type IV (72.0%, 18/25), while few isolates carried type I (12.0%, 3/25), type II (8.0%, 2/25), type III (4.0%, 1/25), and type V (4.0%, 1/25; Supplementary Table S3). MRSA strains belonging to CC22 harbored a lower number of resistance genes compared to the other CCs (Supplementary Figure S1). Besides resistance to β-lactams, the most frequent resistance genes detected in MRSA were aminoglycoside resistance genes (68.0%, 17/25 isolates) followed by macrolide resistance genes (64.0%, 16/25 isolates) and tetracycline resistance genes (16.0%, 4/25 isolates; Table 2). Point mutations conferring resistance to quinolones (*gyrA* and *grlA* genes) and rifampicin (*rpoB* gene) were detected in 80.0% (20/25) and 16% (4/25) of the MRSA isolates, respectively. Discrepancies between the susceptibility phenotype and resistance gene carriage were noticed in few cases for levofloxacin (*n* = 7 isolates), gentamicin (*n* = 2 isolates), rifampicin (*n* = 1 isolate), and daptomycin (*n* = 1 isolate), presumably due to limitations associated with the *in silico* prediction of resistant genes.

## Discussion

*S. aureus* is the most frequent pathogen causing OM, and a wide range of virulence factors involved in adhesion, host cells damage, and evasion of the immune system are likely to account for its success in OM. Given the multifactorial nature of *S. aureus* pathogenesis, this study was aimed at understanding whether *S. aureus* isolates from OM were characterized by (*i*) distinctive epidemiological traits and (*ii*) definite repertoire(s) of virulence-related genes. To this purpose, the clonal characteristics and the virulence determinants inferred from WGS data of 83 *S. aureus* isolates responsible for OM in Italy were investigated. The most frequent CCs were CC22, followed by CC5, CC8 and CC30, which are among the prevalent lineages in Italian nosocomial settings (Giufrè et al., 2017). The phylogenetic relationship based on cgMLST revealed a non-uniform distribution of the isolates that clustered together according to the ST-CC grouping, regardless of the geographic origin or methicillin resistance status. Close genetic relatedness between isolates from either the same or different hospitals was rarely observed, suggesting limited transmission of *S. aureus* strains causing OM within the same hospital and between hospitals in the same geographical area.

The *S. aureus* lineages identified in the present study are largely consistent with those responsible for bone and joint infections worldwide (Pimentel de Araujo et al., 2021), and essentially match the most frequent lineages causing both invasive and non-invasive infections in Italian hospitals (Gagliotti et al., 2012; Grundmann et al., 2014; Giufrè et al., 2017). Although ST8/CC8 is the most common clone involved in OM in several countries (Luedicke et al., 2010; Senneville et al., 2014; Gaviria-Agudelo et al., 2015), the most common lineages in Italy were CC22 and CC5. MSSA isolates showed polyclonality with CC30 being the most represented CC, as previously reported in OM isolates (Post et al., 2014; Valour et al., 2014; Montanaro et al., 2016). MRSA clones belonging to the CC1 and CC8 lineages carried a high number of resistance determinants. Conversely, isolates belonging to CC22, which is one of the most common MRSA lineages in hospital settings in Italy, carried a limited number of resistant genes.

Studies attempting to characterize the virulence of OM strains identified some genetic traits that could be associated with a greater propensity to cause bone infections, such as the *cna* and *bbp* adhesins genes (Post et al., 2014; Montanaro et al., 2016). In our collection *cna* and *bbp* gene were present in 48.2 and 77.1% of the isolates, respectively. In particular, isolates belonging to CC22, the prevalent clone in our collection, showed a wide pattern of adhesin genes, including those coding for collagen adhesin (Cna), bone sialoprotein-binding protein (BbP), and fibronectin binding protein A (FnbA). However, it is worth noticing that each lineage expressed a different combination of adhesin genes, likely reflecting functional redundancy. Previous studies showed how these adhesin genes were frequently associated with invasive infections including bone infections (Palmqvist et al., 2005; Rieg et al., 2013; Foster et al., 2014; Post et al., 2014). Cna has previously been identified in few *S. aureus* clones (Peacock et al., 2002) and has been shown to play an important role in bone tropism in a murine model of haematogenous OM and in the development of septic arthritis (Patti et al., 1994; Elasri et al., 2002). Similarly, BbP is involved in bone tropism and in the initial adhesion to osteoblasts due to its ability to bind bone sialoprotein and fibrogen (Tung et al., 2000; Testoni et al., 2011). FnbA and FnbB have frequently been detected in clinical *S. aureus* isolates (Peacock et al., 2002). FnbA mediates the internalization of *S. aureus* into osteoblasts and adhesion to implant materials enhancing the severity of bone infection and its chronicity (Ahmed et al., 2001; Palmqvist et al., 2005; Testoni et al., 2011; Gries et al., 2020).

After the initial adhesion of the bacteria to bone tissue, the subsequent infection step is biofilm formation. The locus *icaACD* involved in the production of the polysaccharide intercellular adhesin (PIA), which promotes the aggregation of the bacterial cells in biofilm (Heilmann et al., 1996), was detected in over 95% of the *S. aureus* isolates examined in our study. Indeed, the presence of the *ica* locus is very frequent among strains from biofilm-related infection (90%) and less frequent in isolates from carriers (43%; VictoriaMartín-López et al., 2002). Capsular genes were detected in all the isolates, and *cap5* was the most frequent irrespective of the CC. Other important virulence factors, such as the PVL toxin genes, which are a hallmark of community-acquired MRSA (Otto, 2013), were rarely detected, being present in only two CC30 strains. This is probably because OM patients in our study were adults, while PVL genes were more frequently detected in *S. aureus* from paediatric OM (Kechrid et al., 2011; Bouras et al., 2018), and only occasionally in isolates from adults (Senneville et al., 2014; Valour et al., 2014). Other leucocidin genes, such as *lukX* and *lukY*, and haemolysin genes *aur*, *hla*, *hlII* and *hlgA/B/C*, were ubiquitous among the strains. The enterotoxins cluster *seg-sei-sem-sen-seo*, which has been documented to increase the commensal fitness of *S. aureus* (Nowrouzian et al., 2015), was detected in the most frequent lineages CC5, CC22, and CC30. The expression of several virulence genes such as alpha-toxin (*hla*), gamma-hemolysin (*hlg*), leucocidins (*lukX* and *lukY*), and adhesins is under the control of the *agr* system (Cheung et al., 2011; Jenul and Horswill, 2019), which has been directly associated with the pathogenesis of OM (Gillaspy et al., 1995). The *agr* system was detected in nearly 90% of the isolates. In this study, the higher frequency of isolates carrying *agr I* and *agrII*, compared with *agrIII* and *agrIV*, is in accordance with previous reports with other collections of isolates from bone infections (Montanaro et al., 2010; Kawamura et al., 2011).

Studies on the association between *S. aureus* genetic traits and clinical manifestations are challenging and often generated contradictory results. Some studies support that certain *S. aureus* lineages harboring specific sets of virulence genes are more successful than others in causing invasive disease (Rasmussen et al., 2013; Tasse et al., 2018). However, the limit of these studies is the consensus repertoire of virulence genes that are shared within the lineage. Thus, the association of virulence factors and disease can be biased by an uneven distribution of CC between the group of isolates investigated (hitchhiker effect; Lindsay et al., 2006). Strains causing OM show substantial heterogeneity of virulence factors as it is for isolates causing colonization. A recent study showed that the commensal nasal isolates shared the same CC and genetic determinants with isolates from joint infection, suggesting that commensal *S. aureus* clones can cause bone and joint infections (Wildeman et al., 2020). Similarly, there is no evidence that a particular lineage or a single virulence factor or a combination of factors were distinctive of isolates from bone and implant infections (Luedicke et al., 2010) or from invasive infections (van Belkum et al., 2009). Although belonging to several lineages and being characterized by heterogeneous virulence profiles, strains isolated from carriers can become invasive under certain circumstances (Mehraj et al., 2016; Deinhardt-Emmer et al., 2018). Given that *S. aureus* clones causing OM originate from commensal clones which are characterized by an uneven distribution of virulence genes, it is not surprising that *S. aureus* strains from our collection show a broadly diversified pattern of virulence-related traits.

## Conclusion

In summary, this is the first Italian study providing a genome-level characterization of a large collection of *S. aureus* isolates from bone infections. The prevalence of the different CCs in OM follows the epidemiology of *S. aureus* infections in Italy. It is conceivable that the prevalence of certain *S. aureus* lineages in OM is due to their more frequent circulation among patients rather than to a particular pattern of associated virulence factors. Understanding the virulence of *S. aureus* and the consequent infection tropism(s) continues to be a challenging topic for the scientific community. A better understanding of this aspect could be useful for the development of prevention and treatment strategies for OM.

## Data Availability Statement

The datasets presented in this study can be found in online repositories. The names of the repository/repositories and accession number(s) can be found at: https://www.ncbi.nlm.nih.gov/, PRJNA784720.

## Ethics Statement

The studies involving human participants were reviewed and approved by the Ethics committee of Istituto Superiore di Sanità no. 0013802 of 18 April 2019. Patients were required to sign an informed consent that included the acceptance of the collection and analysis of clinical and microbiological data for epidemiologic and scientific purposes. Patients’ data were anonymized.

## Author Contributions

FPA, AP, and PV conceived the study. AP, PV, MM, and MDG contributed to the design of the study. SA, DL, TC, RG, and ER provided the isolates and patients’ data. FPA performed the experiments and drafted the manuscript. FPA, MP, and MDG performed the WGS. FPA and MP analysed the data. FPA, MP, AP, PV, MDG, and MM revised the manuscript. All authors read and approved the final version of the manuscript.

## Funding

This work was supported in part by the Excellence Departments grant (art. 1, commi 314–337 Legge 232/2016) to the Department of Science, Roma Tre University, and grant PRIN 2017 (Prot. 20177J5Y3P) to PV, both from the Italian Ministry of Education, University and Research (MIUR), and in part by the Italian Ministry of Health, Centro Controllo Malattie (CCM), 2019 project “Sostegno alla Sorveglianza delle Infezioni correlate all’assistenza anche a supporto del PNCAR.”

## Conflict of Interest

The authors declare that the research was conducted in the absence of any commercial or financial relationships that could be construed as a potential conflict of interest.

## Publisher’s Note

All claims expressed in this article are solely those of the authors and do not necessarily represent those of their affiliated organizations, or those of the publisher, the editors and the reviewers. Any product that may be evaluated in this article, or claim that may be made by its manufacturer, is not guaranteed or endorsed by the publisher.

## Footnotes

^1^ https://www.eucast.org/clinical_breakpoints/

^2^ https://w3.iss.it/site/aries/

^3^ http://www.genomicepidemiology.org/

^4^ https://pubmlst.org

^5^ https://github.com/tseemann/abricate
