# Supplementary material for: Virulence Determinants in Staphylococcus aureus Clones Causing Osteomyelitis in Italy
Source: Front Microbiol. 2022 Mar 3;13:846167. doi: 10.3389/fmicb.2022.846167 (PMC8927738; doi:10.3389/fmicb.2022.846167)
Supplement: Supplementary file 2 [file Image_1.PDF]

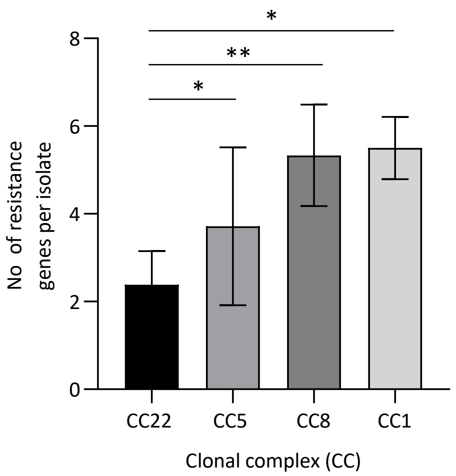

FIGURE S1. Distribution of resistance genes for non ß-lactams antibiotics in MRSA isolates according to clonal complex (CC). CC22, n=13; CC5, n=7; CC8, n=3; CC1, n=2. *, P <0.05; **, P < 0.01.
